# Structural and functional validation of a highly specific Smurf2 inhibitor

**DOI:** 10.1002/pro.4885

**Published:** 2024-02-01

**Authors:** Tanner M. Tessier, Arvid Chowdhury, Zane Stekel, Julia Fux, Maria Augusta Sartori, Joan Teyra, Nick Jarvik, Jacky Chung, Igor Kurinov, Frank Sicheri, Sachdev S. Sidhu, Alex U. Singer, Wei Zhang

**Affiliations:** ^1^ Department of Molecular and Cellular Biology University of Guelph Guelph Ontario Canada; ^2^ Department of Molecular Genetics University of Toronto Toronto Ontario Canada; ^3^ Icosagen Cell Factory OÜ Tartumaa Estonia; ^4^ Department of Pharmacy University of Waterloo Kitchener Ontario Canada; ^5^ NE‐CAT, Department of Chemistry and Chemical Biology Cornell University Argonne Illinois USA; ^6^ Lunenfeld‐Tanenbaum Research Institute, Mount Sinai Hospital Toronto Ontario Canada

**Keywords:** crystal structure, E3 ligases, HECT domain, inhibitor, phage display, protein engineering, ubiquitin variants

## Abstract

Smurf1 and Smurf2 are two closely related member of the HECT (homologous to E6AP carboxy terminus) E3 ubiquitin ligase family and play important roles in the regulation of various cellular processes. Both were initially identified to regulate transforming growth factor‐β and bone morphogenetic protein signaling pathways through regulating Smad protein stability and are now implicated in various pathological processes. Generally, E3 ligases, of which over 800 exist in humans, are ideal targets for inhibition as they determine substrate specificity; however, there are few inhibitors with the ability to precisely target a particular E3 ligase of interest. In this work, we explored a panel of ubiquitin variants (UbVs) that were previously identified to bind Smurf1 or Smurf2. In vitro binding and ubiquitination assays identified a highly specific Smurf2 inhibitor, UbV S2.4, which was able to inhibit ligase activity with high potency in the low nanomolar range. Orthologous cellular assays further demonstrated high specificity of UbV S2.4 toward Smurf2 and no cross‐reactivity toward Smurf1. Structural analysis of UbV S2.4 in complex with Smurf2 revealed its mechanism of inhibition was through targeting the E2 binding site. In summary, we investigated several protein‐based inhibitors of Smurf1 and Smurf2 and identified a highly specific Smurf2 inhibitor that disrupts the E2–E3 protein interaction interface.

## 1 IMPORTANCE

The ubiquitin‐proteasome system is a key regulator of cellular homeostasis, and perturbations in this system are frequently observed in disease. Targeted inhibition of this system by disrupting protein–protein interactions between an E3 ligase and E2 conjugating enzyme has largely been unsuccessful with small‐molecule inhibitors. Here, we identified a ubiquitin variant that specifically inhibits the human E3 ligase Smurf2 by disrupting the E2–E3 interaction.

## 2 INTRODUCTION

Post‐translational modification of proteins by ubiquitination allows for the dynamic regulation of protein activity and abundance across a range of biological processes (Damgaard, 2021). One of the major pathways regulating protein abundance is the ubiquitin‐proteasome system (UPS), where proteins marked with ubiquitin can be mostly directed to the proteasome for degradation. Beyond degradation, ubiquitination plays a pivotal role in numerous cellular processes such as subcellular localization, transcription, DNA damage, metabolism, and the antiviral immune response (Al‐Hakim et al., 2010; Shen et al., 2021; Sulkshane et al., 2020; Trotman et al., 2007; Yao & Ndoja, 2012). Dysregulation of ubiquitin signaling is implicated in a variety of diseases, including cancer and neurodegeneration, and is a common target of pathogens (Deng et al., 2020; Schmidt et al., 2021; Viswanathan et al., 2010). Consequentially, the breadth of ubiquitin signaling has made the UPS a broad therapeutic target with enormous potential (Hoeller & Dikic, 2009; Huang & Dixit, 2016; Schmidt et al., 2021).

Ubiquitination occurs through an enzymatic cascade comprised of a ubiquitin‐activating enzyme (E1), ubiquitin‐conjugating enzyme (E2), and a ubiquitin‐ligase enzyme (E3) (Komander & Rape, 2012). Following activation of ubiquitin by an E1 enzyme, of which only two have been identified in humans; ubiquitin is transferred to one of the several dozen E2 enzymes. The transfer of ubiquitin from an E2 to the appropriate substrate is facilitated by one of several hundred E3 ligases, which provide target substrate specificity. Addition of ubiquitin predominantly occurs on the ε‐amine of lysine residues or the primary amine of a protein substrate's N‐terminus; however, other residues such as serine, threonine and cysteine may also be ubiquitinated (Tait et al., 2007; Vosper et al., 2009). Additional complexity within the ubiquitin signaling pathway comes from the formation of unique polyubiquitin chains. Ubiquitin itself can undergo ubiquitination on a number of lysine residues, which form distinct chains that govern the outcome of a given substrate (Komander & Rape, 2012). Currently, the human genome is expected to encode for more than 800 E3 ligases that can be categorized into four different classes: HECT (homology to E6‐AP carboxy terminus), RING‐finger (Really Interesting New Gene), U‐box, and RBR (RING‐in‐between‐RING) (Kwon & Ciechanover, 2017; Yang et al., 2021). The large number of E3s, compared to E1 and E2 enzymes, allows for tight regulation of protein activity and/or abundance across diverse cellular processes and subcellular localizations (Grabbe et al., 2011).

Small‐molecule inhibitors targeting the UPS have been tested against each enzymatic component of the ubiquitin cascade, with the majority of inhibitors targeting the proteasome directly (X. Zhang et al., 2020). Targeting either the proteasome, E1s, or E2s have a much broader and non‐specific effect compared to E3s, which are responsible for providing target specificity within the UPS (Huang & Dixit, 2016; Landré et al., 2014). Therefore, a rational approach to developing inhibitors would involve disrupting the E2–E3 interaction by specifically targeting the E3 ligase. While the different classes of E3 ligases may differ in how they transfer ubiquitin from an E2 to a substrate, the E2–E3 interaction is a conserved event. Indeed, masking the E2 interaction surface of an E3 is a mechanism already employed by cells to regulate the activity of E3s (Duda et al., 2012). Exploiting this therapeutically would require disrupting a protein–protein interaction (PPI), which has largely been unsuccessful with small molecules due to the typically large and flat interaction surfaces formed between interacting proteins (Lu et al., 2020).

Previously, we have established a structure‐based protein engineering pipeline to systematically develop ubiquitin variants (UbVs) as modulators of E3 ligases (LeBlanc et al., 2021; W. Zhang et al., 2016). By subjecting a wild‐type ubiquitin molecule to directed evolution using phage display, we have generated high‐affinity UbV binders against a variety of E3 ligases (W. Zhang et al., 2016). Depending on their mode of binding, these UbVs have been shown to act as inhibitors or allosteric activators. Structural analysis of several of these inhibitors demonstrated their occupancy at the E2‐binding site, highlighting their ability to disrupt the E2–E3 PPI interface.

With this strategy, we previously identified several UbVs with the ability to target the highly homologous E3 ligases Smurf1 and Smurf2, which belong to the NEDD4 subfamily of HECT ligases (Chen & Matesic, 2007; W. Zhang et al., 2016). At the protein level, Smurf1 and Smurf2 share >70% sequence identity, have high structural similarity and regulate the proteasomal degradation of numerous proteins (Koganti et al., 2018). Smurf1 and Smurf2 were originally identified as important modulators of the bone morphogenetic protein (BMP) and transforming growth factor‐beta (TGF‐β) pathways (Fu et al., 2020). Additionally, these proteins are important contributors to both tumorigenesis and tumor suppression, and altered expression has been observed in several cancer types (David et al., 2013; Fu et al., 2020). Identification of specific Smurf inhibitors, and E3 ligase inhibitors in general, that disrupt the E2–E3 interaction via the E2‐binding site remain underexplored. Such inhibitors would be an invaluable tool for dissecting the molecular roles of Smurf1 and Smurf2, and other E3 ligases.

Here, we first examined several previously identified UbVs with the ability to inhibit Smurf1 and Smurf2 E3 ligase activity. Using in vitro and cell‐based assays, we identified a highly specific Smurf2 targeting UbV, UbV S2.4, that bound and inhibited Smurf2 with nanomolar affinity. Remarkably, UbV S2.4 did not show any cross‐reactivity toward Smurf1. Structural analysis of UbV S2.4 in complex with Smurf2 demonstrated its mechanism of action involves targeting the E2‐binding surface. Overall, these results demonstrate the potential of UbVs to specifically disrupt the E2–E3 PPI interface by directly targeting the E2‐binding site.

## 3 RESULTS

### 3.1 In vitro specificity analysis of Smurf1 and Smurf2 UbV inhibitors

**FIGURE 1:**
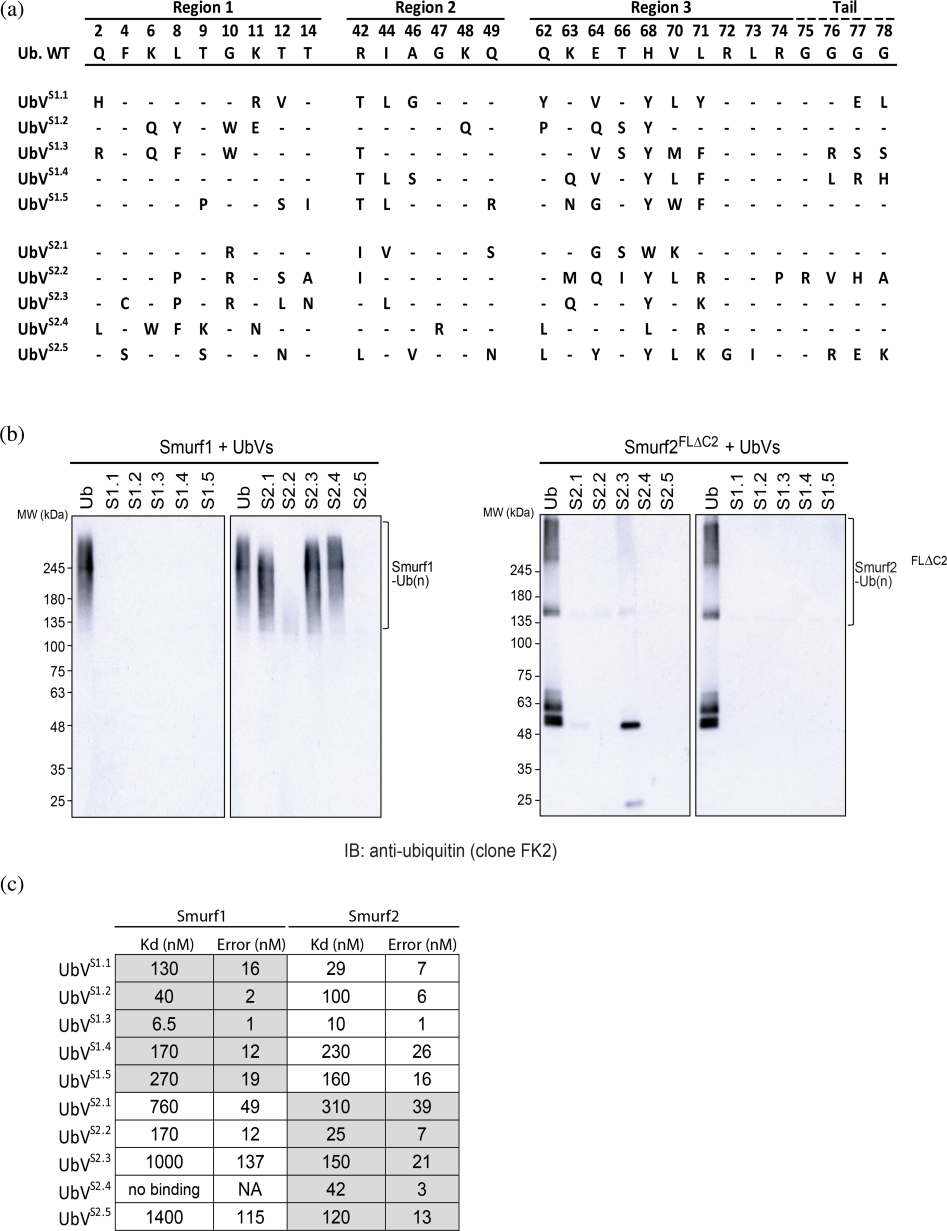
Ubiquitin variant (UbV) selection and specificity analysis. (a) UbVs generated against Smurf1 (UbV S1.1–5) and Smurf2 (UbV S2.1–5) by phage display. Regions 1, 2 and 3 correspond to regions of wild‐type ubiquitin that were subjected to diversification. Sequence differences, relative to wild‐type ubiquitin, for each UbV are indicated. (b) Specificity of UbVs assessed by in vitro auto‐ubiquitination assays using purified full‐length Smurf1 (left) or Smurf2 lacking the autoinhibitory C2 domain (Smurf2FLΔC2; right). (c) Affinities of UbVs for full‐length Smurf1 and Smurf2 proteins were determined by biolayer interferometry assays.

Previously, we performed phage display using UbV libraries targeting members of the HECT E3 ligase family (W. Zhang et al., 2016). This included UbVs generated against full‐length Smurf1 and Smurf2 lacking the autoinhibitory C2 domain (Smurf2FLΔC2). This resulted in the identification of several Smurf1 and Smurf2 UbVs with the potential to function as highly specific Smurf modulators. Inspection of wild‐type ubiquitin and the corresponding regions that were subjected to diversification (regions 1, 2 and 3) demonstrated considerable variability between Smurf1 and Smurf2 UbVs (Figure 1a). Moreover, the majority of diversified positions showed limited sequence similarity between groups, potentially giving rise to UbVs with highly specific binding properties.

Given the high sequence and structural similarity between Smurf1 and Smurf2, there is potential for cross‐specificity toward either Smurf HECT domain. Using in vitro auto‐ubiquitin assays, we previously showed that Smurf1 and Smurf2 UbVs are able to inhibit their cognate Smurfs (W. Zhang et al., 2016); however, their cross‐specificity using a functional assay was not assessed. To determine this, we similarly performed in vitro auto‐ubiquitination assays using Smurf1 and Smurf2FLΔC2 (Figure 1b). Interestingly, all UbVs generated against Smurf1 (S1.1–1.5) also exhibited potent inhibition of Smurf2 HECT ligase activity. In contrast, UbVs generated against Smurf2 had a variable effect on Smurf1 HECT ligase activity. UbVs S2.2 and S2.5 demonstrated clear cross‐reactivity with Smurf1 and potently inhibited Smurf1 ligase activity. UbV S2.1 and S2.3 appeared to have a potential modulatory effect based on the pattern of lower molecular weight ubiquitinated Smurf1 compared to the wild‐type ubiquitin control. Most notable was UbV S2.4, which had no obvious effect on Smurf1 HECT ligase activity and may represent an ultra‐specific Smurf2 inhibitor.

To further assess the binding affinity and cross‐specificity of these UbVs, we utilized bio‐layer interferometry (BLI) to quantitatively determine binding kinetics using full‐length Smurf1 and Smurf2 proteins (Figure 1c). For UbVs targeting Smurf1, S1.3 exhibited the highest affinity (*K*_*d*_ < 10 nM), but affinity for Smurf2 was nearly identical, agreeing with the cross‐specificity observed using auto‐ubiquitination assays. In fact, all UbVs generated against Smurf1 show relatively high binding affinities for Smurf2 (*K*_*d*_ < 250 nM), some of which have a higher affinity for Smurf2 than Smurf1, such as S1.1 and S1.5. Interestingly, Smurf2 UbVs also demonstrated high affinities, but unlike Smurf1 UbVs, these consistently showed higher affinities for Smurf2 compared to Smurf1. Consistent with what we observed using auto‐ubiquitination assays, we did not observe any detectable binding between UbV S2.4 and Smurf1.

Overall, phage display yielded several UbVs with the ability to bind Smurf1 and/or Smurf2 with high affinity. Moreover, we possibly identified cross‐reactive UbVs, which may potentially be utilized as pan‐Smurf inhibitors. These results also yielded an ultra‐specific UbV that can selectively bind and inhibit Smurf2 but not Smurf1 in vitro.

### 3.2 UbV S2.4 is a specific binder and inhibitor of Smurf2 in cells

**FIGURE 2:**
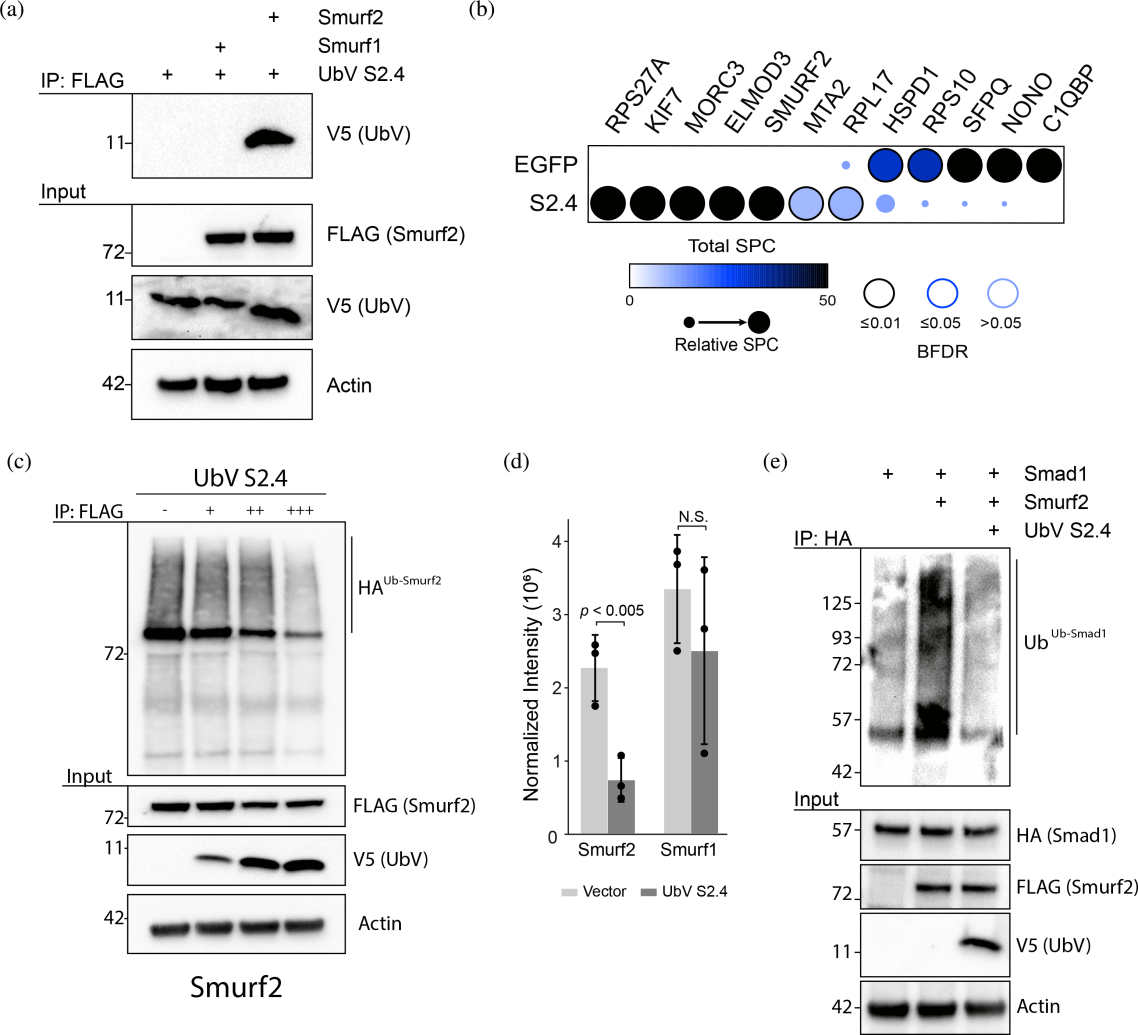
Specificity analysis of UbV S2.4 in cells. (a) Co‐immunoprecipitation of UbV S2.4 with Smurf1 and Smurf2. HEK293 cells were transfected with either FLAG‐Smurf1 or FLAG‐Smurf2 and V5‐tagged UbV S2.4. Immunoprecipitations using FLAG were performed and coimmunoprecipitation of UbV S2.4 was determined using anti‐V5 antibody. (b) Affinity purification‐mass spectrometry analysis of UbV S2.4 interactors. HEK293T cells were transfected with FLAG‐tagged UbV S2.4, FLAG‐EGFP, or an empty vector control. Spectral counts (SPC) were used to identify significant interactors relative to the control sample using SAINTexpress. Proteins with a Bayesian false‐discovery rate (BFDR) ≤0.05 are deemed significant compared to the empty vector control. (c) Smurf2 auto‐ubiquitination assay. HEK293 cells were transfected with FLAG‐tagged Smurf2 and HA‐tagged wild‐type ubiquitin and a 0.5:1 (+), 1:1 (++) or 2:1 (+++) ratio of V5‐UbV S2.4 to Smurf2. Cells were treated with 40 μM MG‐132 prior to anti‐FLAG immunoprecipitation to enrich Smurf1 or Smurf2. Immunoblotting with anti‐HA antibody was used to determine the level of auto‐ubiquitinated Smurf2 protein. See Figure S1A for Smurf1 auto‐ubiquitination. (d) Quantitative western blotting of UbV S2.4 mediated inhibition of Smurf1 and Smurf2. Auto‐ubiquitination assays were performed as outlined in panel (c) using 2:1 ratio of V5‐UbV S2.4 to Smurf1 and Smurf2. Student's *t* test was used to determine statistical significance. *n* = 3. See Figure S1B for western blots used for quantification. (e) UbV S2.4 mediated inhibition of Smad1 ubiquitination. HEK293 cells were transfected with Smad1‐HA, FLAG‐Smurf2, and V5‐UbV S2.4, treated with 10 μM MG132 and immunoprecipitated using anti‐HA. Smad1 ubiquitination levels were assessed using anti‐ubiquitin antibody against endogenous ubiquitin.

In vitro binding experiments demonstrated UbV S2.4 may represent an ultra‐specific Smurf2 inhibitor. To determine if this specificity is observed within a complex cellular environment, we first performed co‐immunoprecipitation experiments (Figure 2a). HEK293 cells were transfected with V5‐tagged UbV S2.4 and either FLAG‐tagged Smurf1 or Smurf2. Anti‐FLAG immunoprecipitation followed by anti‐V5 immunoblotting was performed to assess binding of UbV S2.4 to Smurf1 and Smurf2. Immunoprecipitation of Smurf1 and Smurf2 demonstrated UbV S2.4 is specific toward Smurf2 as there was no detectable interaction with Smurf1. These data agree with the in vitro binding assays demonstrating UbV S2.4 is specific for Smurf2.

To further assess UbV S2.4 specificity within a cellular context and in an unbiased manner we performed affinity purification‐mass spectrometry (AP‐MS) (Figure 2b). HEK293T cells were transfected with either FLAG‐UbV S2.4, FLAG‐EGFP, or an empty vector control, and FLAG immunoprecipitations were performed. Identification of significant protein interactors compared to the empty vector control was determined by processing spectral count data using SAINTexpress and visualized with ProHits‐viz (Knight et al., 2017; Teo et al., 2014). Among the enriched interactors for UbV S2.4 were Rps27A, Rpl17 and Mta2, which most likely represent background contaminants as spectral counts/peptides corresponding to these proteins are frequent among AP‐MS experiments (Mellacheruvu et al., 2013). In contrast, Kif7, Morc3, Elmod3, and Smurf2 were identified as significant interactors, suggesting these may be bona fide binding proteins of either Smurf2 or UbV S2.4. Most importantly, we did not detect any evidence of other E3 ligases, including Smurf1, or E1/E2 enzymes within the dataset, further demonstrating that UbV S2.4 is specific for Smurf2.

To determine the inhibitory potential of UbV S2.4 within a cellular context, we next assessed the ability of UbV S2.4 to inhibit Smurf1 and Smurf2 E3 ligase activity through cellular auto‐ubiquitination assays, as was similarly done by Tian et al. (2019) (Figure 2c and S1A). Briefly, HEK293 cells were transfected with full‐length FLAG‐Smurf1 or FLAG‐Smurf2, HA‐tagged wild‐type ubiquitin, and increasing amounts of UbV S2.4. Cells were treated with the proteasome inhibitor MG‐132 and FLAG immunoprecipitations were performed to enrich Smurf1 or Smurf2 in order to assess their degree of auto‐ubiquitination. In agreement with the in vitro ubiquitination assays (Figure 1b), UbV S2.4 was able to function as a Smurf2 inhibitor within a cellular context, and this was observed to be true in a dose‐dependent manner (Figure 2c). When assessing cross‐reactivity, we observed weak inhibition of Smurf1 auto‐ubiquitination in the presence of UbV S2.4; however, the degree of inhibition was notably lower than what was observed with Smurf2 (Figure S1A). Furthermore, quantitative western blotting of Smurf1 and Smurf2 auto‐ubiquitination in the presence of UbV S2.4 demonstrated a significant reduction in Smurf2 ubiquitination but not Smurf1 (Figure 2d and S1B). While not significant, the observed decrease in Smurf1 auto‐ubiquitination occurred with an increase in Smurf1 input protein levels (Figure S1A). Furthermore, co‐expression of FLAG‐Smurf2 with MYC‐Smurf1 demonstrated Smurf2‐mediated degradation of Smurf1, and this could be inhibited by the addition of UbV S2.4 (Figure S1C). These results strongly suggest that inhibition of endogenous Smurf2 in these conditions likely explains the decrease in transfected FLAG‐Smurf1 ubiquitination levels.

To determine if inhibition of Smurf2 has downstream effects on known Smurf2 substrates, we performed Smad1 ubiquitination assays. Briefly, HEK293 cells were transfected with a combination of Smad1‐HA, FLAG‐Smurf2, and V5‐UbV S2.4 and followed by immunoprecipitation of Smad1 to directly evaluate ubiquitination (Figure 2e). As expected, co‐expression of Smad1‐HA and FLAG‐Smurf2 increased Smad1 ubiquitination. Upon addition of UbV S2.4, Smad1 ubiquitination levels were substantially reduced. Overall, these observations demonstrate that UbV S2.4 is able to specifically bind and inhibit Smurf2 E3 ligase activity within a cellular context.

### 3.3 Structural analysis of UbV S2.4 bound to Smurf2

**FIGURE 3:**
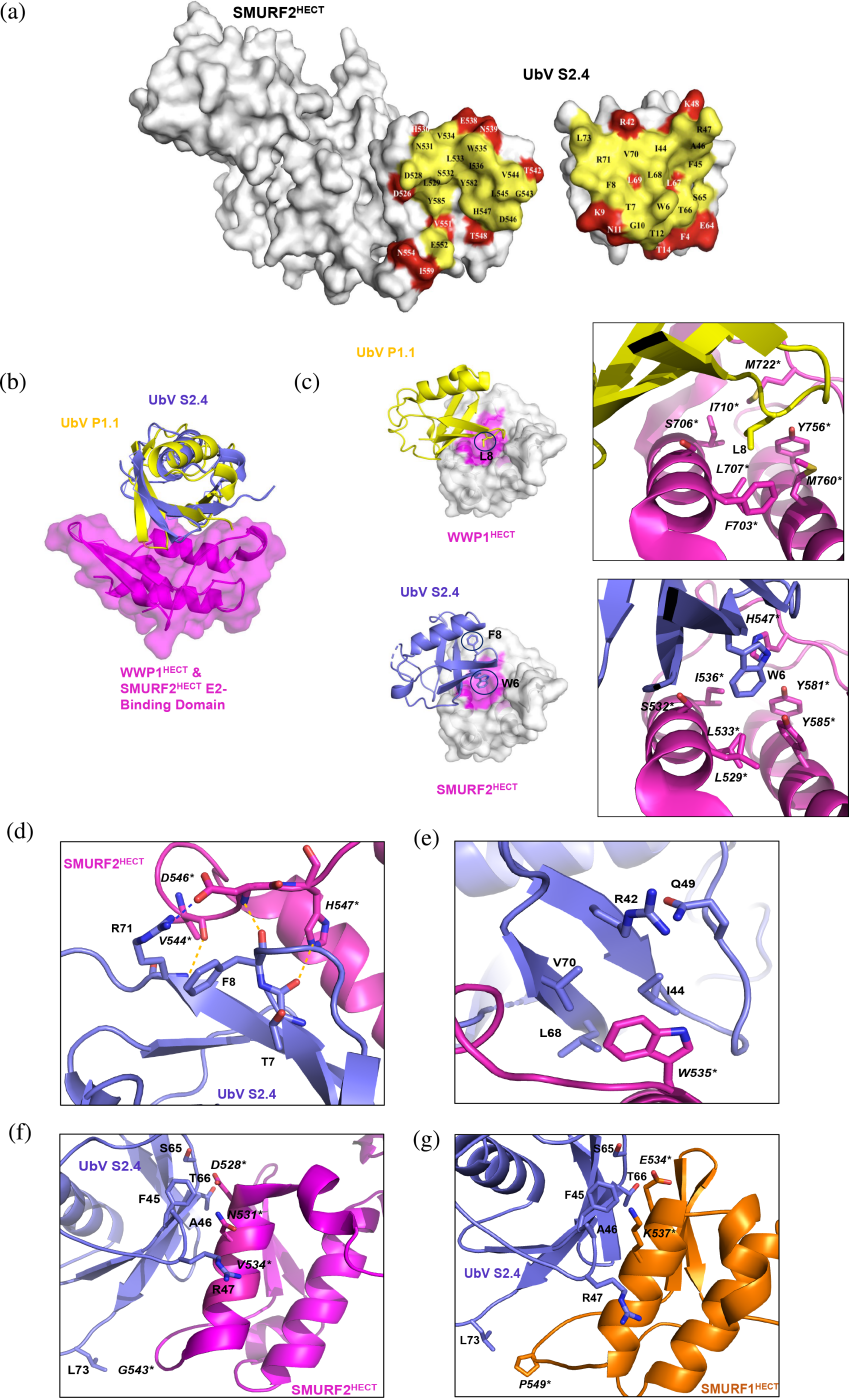
Structural analysis of the Smurf2 HECT domain in complex with UbV S2.4. (a) Open book view of the complex with the HECT domain of Smurf2 (left panel) and UbV S2.4 (right panel) shown as molecular surfaces. Contacting residues are colored yellow, non‐contacting residues are colored white and weakly bound (<20% buried upon formation of the complex) are colored red. (b, c) Structural comparison of UbV S2.4 bound to the Smurf2 HECT domain and UbV P1.1 bound to the WWP1 HECT domain (PDB ID: 5HPS), with UbV P1.1 and S2.4 colored yellow and blue, respectively. HECT residues are denoted with an asterisk to distinguish them from UbV residues. (b) Position of UbVs following superposition of the E2‐binding sites of the two HECT domains. The E2‐binding domain of Smurf2 (residues 516–593) is shown as a transparent surface and HECT E2‐binding region and superimposed UbVs are shown as cartoons. (c) Two views of the hydrophobic pocket of the WWP1 (top panels) and Smurf2 (bottom panels) HECT domains in complex with their UbVs. In the left panels, the HECT domains are shown as transparent molecular surfaces, with residues contacting L8 and W6 colored pink and shown as sticks, and L8 of WWP1 and W6 and F8 shown as sticks are highlighted with a circle. In the right panels, a closeup of L8 of UbV P1.1 (top), and W6 of UbV S2.4 (bottom), and their contacting residues are shown. (d, e) Closeup of two additional regions. (d) Interactions of the UbV with a loop in Smurf2 (residues 544–547), in which a number of hydrogen bonds and a salt bridge are observed (yellow dashes represent hydrogen bonds, the blue dash represents the salt bridge between R72 of the UbV and D546 of Smurf2). (e) UbV S2.4 residues contacting Smurf2 W535. (f, g) Basis for the specificity of UbV S2.4 for Smurf2 versus Smurf1. (f) The three residues in Smurf2 that interact with UbV S2.4 and differ from Smurf1 are shown and labeled, as well as their contacting residues in UbV S2.4. (g) A structural model of the Smurf1 HECT domain (orange) in complex with UbV S2.4 built using SWISS‐MODELLER. The three residues in Smurf1 that interact with UbV S2.4 and differ from Smurf2 are shown and labeled, as well as their putative contacting residues in UbV S2.4.

The potential utility of UbV S2.4 as an ultra‐specific Smurf2 inhibitor was of particular interest, and thus, we wanted to elucidate the structural basis for this interaction. To do so, we determined the crystal structure of the Smurf2 HECT domain in complex with UbV S2.4 at a resolution of 2.5 Å (Table 1). Molecular replacement using PHASER demonstrated little conformational change. In particular, while orientation of the N‐ and C‐lobes of HECT domains are an important source of flexibility (Verdecia et al., 2003), the HECT domain of Smurf2 in this complex appears to adopt the same orientation as in the unbound Smurf2 structure (Figure S2A), with an overall RMSD for the C*α* atoms of 1.7 Å between the HECT domains in the two structures. Furthermore, as one might expect for an inhibitor, UbV S2.4 binds Smurf2 exclusively at the E2‐binding region. In forming this complex, 720 and 747 Å of surface area are buried (Krissinel & Henrick, 2007) on the UbV and HECT domain, respectively (Figure 3a).

**TABLE 1:** Crystal structure for Smurf2HECT in complex with UbV S2.4.

PDB ID	7M3Q
Data collection	
Beamline	APS beamline 24‐ID‐E
Wavelength (Å)	0.97918
Crystals	Native
Unit cell parameters	
Space group	C2
*a*, *b*, *c* (Å)	201.5, 72.1, 48.8
*α*, *β*, *γ* (°)	90.00, 94.6, 90.00
Resolution (Å)	67.83–2.45 (2.55–2.45)
Unique reflections^a^	24,895 (2871)
Completeness (%)	97.1 (99.2)
*R*_pim_	0.084 (0.569)
Overall *I/*𝜎*I*	8.8 (2.2)
Multiplicity	3.3 (3.5)
CC (1/2)	0.982 (0.478)
Wilson *B* factor	54.49
Refinement	
Resolution (Å)	67.83–2.50
*R*_work_ */R*_free_	0.203/0.235
RMSD bond length (Å)	0.009
RMSD bond angle (°)	1.14
No. of protein atoms	3615
No. of water atoms	54
B‐factor average	70.60
B‐factor protein	70.75
Ramachandran statistics (MolProbity)	
Preferred (%)	95.46
Allowed (%)	4.54
Disallowed (%)	0
Clashscore	8.25

^a^ Values in parentheses represent the highest‐resolution shell.

We previously reported the structures of two inhibitory UbVs in complex with their respective HECT domains, namely UbV IT.2 in complex with ITCH (PDB ID: 5C7M) and UbV P1.1 in complex with WWP1 (PDB ID: 5HPS) (W. Zhang et al., 2016). These UbVs bound to their HECT domains in essentially the same manner, with RMSDs of 1.04 Å upon superposition of Cα positions of the coordinates of the HECT E2‐binding region and UbV together. In the Smurf2 structure (Figure 3b), UbV S2.4 binds in a similar binding site but is oriented by a rotation of approximately 20 degrees relative to UbV P1.1. A closer look at the complexes clarifies the reason for these differences (Figure 3c). In the WWP1 structure, the loop between strands β1 and β2 of UbV P1.1 points toward a hydrophobic pocket at the center of the E2‐binding lobe of the HECT domain, with residue L8 in this loop forming numerous hydrophobic contacts with F703*, L707*, I710*, Y756* and M760* of the WWP1 HECT domain (Figure 3c, upper panel). In the case of Smurf2, the hydrophobic pocket formed by the corresponding residues, namely L529*, S532*, L533*, I536*, H547*, Y581* and Y585*, is instead occupied by W6 in strand β1 of UbV S2.4 and F8 points away from the interface (Figure 3c, lower panel). To accommodate this change in position of the UbV, the β1‐β2 loop must avoid steric clashes, and it does so by pressing against the loop between α5 and β7 of Smurf2 (Figure 3d). This adjustment is accommodated by main‐chain hydrogen bonds between F8 and R71 of UbV S2.4 and D546* and V544* of Smurf2, respectively. Moreover, the main‐chain carbonyl of T7 and side‐chain of R71 in the UbV form a hydrogen bond with the side‐chain of H547* and a salt bridge with the side‐chain of D546* in Smurf2, respectively.

In Smurf2, the indole ring of W535* is buried deep within a hydrophobic pocket in UbV S2.4 formed by the side chains of R42, I44, L68 and V70 (Figure 3e). W535* is completely conserved in the HECT domains of the NEDD4 family and is believed to be involved in recognition of the UbcH4, UbcH5 and Ube2E E2 ligases that contain a serine residue corresponding to S94 in UbcH5B (Kamadurai et al., 2009). Thus, W535* is not responsible for the specificity of UbV S2.4 but contributes to affinity.

The HECT domains of Smurf1 and Smurf2 have substantial sequence homology, and yet, UbV S2.4 only binds Smurf2. Intriguingly, there are only three amino acid differences between the two HECT domains among residues interacting with UbV S2.4 (Figure 3f). Specifically, UbV S2.4 contacts residues D528*, N531*, and G543* in Smurf2, and these correspond to residues E534*, K537*, and P549* in Smurf1, respectively. To understand this specificity in more detail, a Smurf1 HECT/UbV S2.4 model was generated by computational modeling with SWISS‐MODELLER (Waterhouse et al., 2018) using the Smurf2 HECT/UbV S2.4 structure as the starting model (Figure 3g). In the Smurf2 HECT/UbV S2.4 structure, one of the carboxylate oxygens of D528* forms a hydrogen bond with the backbone NH and hydroxyl group of T66 in UbV S2.4. However, if E534* in Smurf1 adopts the same conformation, steric clash with UbV residues occurs, and thus in the Smurf1 HECT/UbV S2.4 model, the carboxyl group extends away from the UbV, preventing it from forming any strong interactions. In addition, N531* in Smurf2 intercalates with the UbV loop that contains residues F45, A46 and R47, but with the corresponding K537* residue in Smurf1, the positively charged side‐chain is close to R47 and charge–charge repulsion would occur. In contrast, the change of G543* in Smurf2 to Pro549* in Smurf1 is not predicted to have a deleterious effect on UbV binding since this residue is at the edge of the interface. Thus, two of the three amino acid differences in the E2 binding sites of Smurf1 and Smurf2 are likely to be differentially recognized by UbV S2.4, allowing it to preferentially recognize Smurf2.

## 4 DISCUSSION

Various avenues have been explored to directly target the UPS, but these approaches tend to lack the high specificity one could achieve by targeting E3 ligases directly. Overall, there is a paucity of specific inhibitors relative to the number of E3 ligases encoded within the human genome. Nearly all small‐molecule inhibitors targeting HECT E3 ligases are directed toward sites other than the E2‐binding region, such as the catalytic cysteine, regulatory regions or substrate recognition surfaces (Weber et al., 2019). In fact, very few E2‐binding site inhibitors have been identified thus far. Previously, our group has developed UbVs against a number of HECT E3 ligase family members, some of which were demonstrated to bind E2 binding sites and inhibit E3 ligase activity (W. Zhang et al., 2016). Here, we evaluated several UbV inhibitors of the Smurf1 and Smurf2 HECT E3 ligases. UbV S2.4 was found to be an ultra‐specific Smurf2 inhibitor that did not show any cross‐reactivity toward Smurf1 either in vitro or within a cellular context.

Structural analysis of UbV S2.4 in complex with Smurf2 confirmed that it bound the E2‐binding site and revealed the molecular basis for its specificity. Previously, others have used phage display to generate bicyclic peptides that targeted the E2 binding site on Smurf2 with affinities in the micromolar range (Mund et al., 2014). In contrast, our UbV exceeded those affinities by three orders of magnitude, and these high affinities can be attributed to the extensive, complementary buried surface area at the UbV:Smurf2 interface. Unlike many small molecule inhibitors, UbVs also demonstrated remarkable specificity. In particular, UbV S2.4 bound Smurf2 with a *K*_*d*_ of 42 nM and exhibited no detectible binding to the highly homologous Smurf1 or any other NEDD4 family member (or any other E3 ligase, for that matter). The structure showed that only 2 of the 25 Smurf2 residues that contacted UbV S2.4 differed in Smurf1, but these subtle differences were sufficient to enable high specificity for Smurf2.

These findings highlight the exceptional ability of UbVs to target the E2 binding interface and overcome current limitations of small molecules. While small molecule‐mediated inhibition of PPIs has been improving, the typically large and flat features of PPI interfaces remain problematic (Lu et al., 2020). For example, a small molecule displacement screen of a bicyclic peptide that bound the E2 binding site of Smurf2 failed to identify any compounds that displaced the bicycle by directly binding the E2 binding site (Mund et al., 2014). In the case of highly similar interfaces, such as with the Smurf1 and Smurf2 E2 binding site, small molecule mediated inhibition is unlikely to satisfy specificity requirements. Therefore, utilizing a larger surface area is likely required to achieve sufficient specificity and affinity, which has been demonstrated with UbV‐based inhibitors previously and now here with UbV S2.4 (W. Zhang et al., 2016).

Cellular auto‐ubiquitination assays support the E2‐binding site model for UbV S2.4. HECT E3 ligases form an intermediate covalent bond with ubiquitin (Sluimer & Distel, 2018) and inhibiting this interaction should reduce the formation of HECT domain‐ubiquitin intermediates. The observed dose‐dependent decrease in ubiquitinated Smurf2 upon transfection of UbV S2.4 in cells supports this hypothesis. Moreover, inhibition of Smurf2 was also observed to reduce ubiquitination of Smad1, a well‐established Smurf2 substrate (Y. Zhang et al., 2001). Cross‐specificity analysis in cells using an auto‐ubiquitination assay suggests UbV S2.4 may have a weak ability to inhibit Smurf1; however, this is likely related to UbV S2.4 mediated inhibition of endogenous Smurf2. In support of this, Smurf2 has been demonstrated to regulate Smurf1 activity via ubiquitin‐mediated degradation (Fukunaga et al., 2008). Here, we observed an increase in FLAG‐Smurf1 levels with increasing UbV S2.4 and also show that Smurf1 degradation can be mediated by co‐expression of Smurf2, and subsequently inhibited using UbV S2.4. Given the overall lack of evidence supporting an interaction between UbV S2.4 and Smurf1, decreased Smurf1 auto‐ubiquitination is presumably due to inhibition of endogenous Smurf2.

Co‐immunoprecipitation assays and AP‐MS demonstrated remarkable specificity for Smurf2. Intriguingly, AP‐MS may have also identified Elmod3, Morc3 and Kif7 as novel Smurf2 interactors. Smurf2 has well established roles in cellular migration and invasion, processes which prominently feature regulation of the cytoskeleton (Koganti et al., 2018). Notably, Elmod3 and Kif7 have also been associated with cytoskeleton remodeling or localization (Haque et al., 2022; Xu & Jin, 2019), supporting the possibility that these are cofactors and/or substrates of Smurf2. Moreover, Smurf1 and Smurf2 are recognized regulators of bone homeostasis through the modulation of BMP signaling (Koganti et al., 2018). The identification of Morc3, which has also been associated with bone homeostasis, may also represent another association with Smurf2 (Jadhav et al., 2016). While these interactions require further investigation, their identification highlights the utility of UbVs as an important tool to explore E3 ligase functions.

The highly similar Smurf1 and Smurf2 proteins belong to the NEDD4 subfamily of HECT E3 ligases and have distinct and overlapping roles in a range of biological processes and diseases (Koganti et al., 2018; Wang et al., 2023). Intriguingly, within the context of cancer, these proteins appear to have unique roles as either tumor suppressors or oncogenes, with Smurf2 possibly behaving as both depending on context (Fu et al., 2020). These details highlight the need for highly specific inhibitors in order to dissect their roles more precisely with respect to both disease and their basic biological functions. While intracellular delivery of engineered proteins for therapeutic purposes is a current limitation, UbV S2.4 will be a valuable tool for differentiating the biological roles of Smurf2 and Smurf1. Moreover, several other UbVs we tested, but did not fully characterize, may also serve as valuable tools for investigation Smurf1 and Smurf2, in addition to UbV S2.4.

In conclusion, we were able to identify and characterize an ultra‐specific UbV inhibitor of Smurf2. The mechanism of action was determined through structural analysis of UbV S2.4, which revealed that UbV S2.4 targets the E2‐binding site to block the transfer of ubiquitin. Given the paucity of E2‐binding site inhibitors in general, UbV S2.4 may prove to be a useful tool for studying the role of Smurf2 protein in a variety of cellular contexts.

## 5 MATERIALS AND METHODS

### 5.1 Cell culture, transfections and antibodies

HEK293 cells were grown in high glucose DMEM (Cytiva) supplemented with 1 mM sodium pyruvate, 1× antibiotic‐antimycotic reagent and 10% fetal bovine serum (FBS). Cells were kept in a 5% CO_2_, 37°C incubator and tested for mycoplasma presence. Transfections were performed using the standard Lipofectamine 3000 (Thermo Fisher) protocol. Western blots were performed using the following antibodies unless otherwise specified: polyclonal V5‐HRP (Novus Biologicals), monoclonal FLAG (M2, MilliporeSigma), monoclonal HA (2–2.2.14, Thermo Fisher), monoclonal beta‐actin (AC‐15, Thermo Fisher), MYC (A21281, Thermo Fisher), monoclonal ubiquitin (FK2, MilliporeSigma) and monoclonal ubiquitin (P4D1, Thermo Fisher).

### 5.2 Co‐immunoprecipitations

HEK293 cells were grown on 10‐cm dishes and transfected with the indicated plasmids. Cells were lysed in NP‐40 lysis buffer (Thermo Fisher), supplemented with HALT protease inhibitor cocktail (Thermo Fisher) and incubated with anti‐FLAG magnetic beads (MilliporeSigma) or Protein G Dynabeads (Thermo Fisher) and anti‐HA epitope antibody. Immunoprecipitations were carried out at 4°C for 2 h. Bound proteins were eluted by boiling in 2× LDS (Thermo Fisher) supplemented with DTT.

### 5.3 Protein production and purification

Genes encoding for Smurf1, Smurf2, UbV S1.3 and UbV S2.4 were cloned into pET28a vectors. The vectors were transformed into *Escherichia coli* BL21 (DE3) and grown in LB broth on a shaker at 37°C, 200 rpm. Once OD600 of 0.7 was reached, 0.5 mM IPTG was added and the culture was incubated for 12 h at 16°C, 200 rpm. Following incubation, the cells were lysed by sonication. The recombinant proteins were purified using FPLC with a HisTrap column.

### 5.4 Crystallization and structural determination of UbV S2.4 and Smurf2

A gene encoding for Smurf2 was cloned into the pProEX‐HTa vector, and a gene encoding for UbV S2.4 (W. Zhang et al., 2016) was cloned into the pET53 vector, and each was expressed in *E. coli* BL21 (DE3) (New England Biolabs). The proteins were purified as described previously (W. Zhang et al., 2016). Briefly, lysate containing the His‐tagged proteins was applied to a Ni‐NTA column (Qiagen 30250), washed and eluted stepwise using 30 and 400 mM imidazole. The His‐tagged proteins in the high‐imidazole elution were subject to TEV cleavage, and the TEV cleavage digests containing each of the Smurf2 and UbV proteins were mixed, keeping the UbV in molar excess. The protein complex was separated from the excess UbV and 6×His tag using a preparative Superdex Increase HiScale S75 16/40 column (GE Healthcare) and samples from fractions were run on an SDS‐PAGE gel. Fractions containing both Smurf2 and UbV were concentrated to 10 mg/mL in 20 mM HEPES buffer, 150 mM NaCl and 1 mM dithiothreitol for crystallization trials.

Crystallization trials were performed using commercial crystallization screens. Initial hits were subjected to refinement in Cryshem sitting drop plates (Hampton Research). Initial crystallization hits for the Smurf2 HECT/S2.4 UbV complex were either small clusters of needles or overlapping clusters of twinned plates. The N‐terminal 6×His tag on UbV S2.4, which is expected to be unstructured (sequence MAHHHHHHVTSLYKKAGDYKDDK), also contains 4 lysine residues. Upon addition of limiting amounts of trypsin to the HECT/UbV complex, the twinned plate appearance was improved such that individual plates could be observed in the cluster, broken off and picked for data collection (Figure S2B). The protein complex was crystallized in 1.5 M ammonium sulfate and 100 mM MES buffer pH 6.0, and trypsin was added at a final concentration of 10 μg/mL (Dong et al., 2007). The crystals were cryoprotected in a solution of 1.4 M ammonium sulfate, 100 mM MES and 25% ethylene glycol and flash‐frozen in liquid nitrogen prior to data collection.

Diffraction data were collected at beamline NECAT 24‐ID‐E at the Advanced Photon Source (ANL, Argonne, IL). Datasets were collected from a single crystal remotely at 100 K using web‐based remote GUI developed by the NECAT team. Individual datasets were indexed, integrated with XDS (Kabsch, 2010), and scaled with Aimless (Agirre et al., 2023). These data were used to solve the structure with Phenix.Phaser (McCoy et al., 2007) using the structure of the Smurf2 HECT domain (PDB ID: 1ZVD) (Ogunjimi et al., 2005) and a SWISS‐MODELLER model of the UbV as models for molecular replacement of the HECT domain and UbV, respectively. Following molecular replacement, model refinement and water picking were done using iterative cycles of Phenix.refine (including automated water picking and TLS parameterization) (Urzhumtsev et al., 2013) and manual model refinement using the molecular graphics program Coot (Emsley & Cowtan, 2004). The final model showed good geometry with no Ramachandran angle violations. The structure of Smurf2^HECT^ in complex with UbV S2.4 has been deposited in the Protein Data Bank with the identification code 7M3Q.

### 5.5 Biolayer interferometry

BLI experiments were performed as outlined in W. Zhang et al., 2016. In brief, proteins were diluted into BLI reaction buffer (25 mM HEPES pH 7.0, 150 mM NaCl, 0.1 mg/mL bovine serum albumin, 0.01% Tween20) and measurements were collected on an Octet RED96 system (ForteBio; Octet Analysis 9.0 software) using anti‐GST antibody biosensors for GST‐tagged ligands (HECT domains) and His‐tagged analytes or wild‐type ubiquitin at 25°C. Up to nine dilution points of analytes covering a wide concentration range were applied. Dissociation constants (*K*_*d*_) were obtained by fitting the response wavelength shifts in the steady‐state regions using single‐site binding system or non‐equivalent two‐site binding system (see Zhang et al., for equations used). To obtain *R*_max_ and *K*_*d*_ values the Levenberg–Marquardt algorithm was used to perform iterative non‐linear least squares curve fitting in Profit 6.2 (QuantumSoft).

### 5.6 Mass spectrometry and data analysis

Frozen HEK 293T cell pellets from transfected 15‐cm plates were thawed into 1 mL High Salt AFC buffer (10 mM Tris–HCl pH 7.9, 420 mM NaCl, 0.1% NP‐40, 1 mM sodium orthovanadate, 2 mM sodium pyrophosphate, 10 mM NaF, protease inhibitor cocktail [MilliporeSigma S8830]). Cell suspensions were then subjected to 3 freeze–thaw cycles, sonicated (5 cycles of 0.3 s on and 0.7 s off) and incubated for 30 min at 4°C with 12.5–25 U of benzonase nuclease (MilliporeSigma E1014). The samples were centrifuged at 13,000 rpm for 30 min at 4°C and 10 μL slurry of M2 anti‐Flag beads (Sigma 8823) was added for overnight incubation. Beads were washed 2 times with low salt AFC buffer (10 mM Tris–HCL, pH 7.9, 100 mM NaCl, 0.1% NP‐40) and 3 times with low salt AFC buffer without detergent. Immunoprecipitated proteins were eluted from the beads with 0.5 M ammonium hydroxide, 4 × 50 μL for a total of 200 μL. Samples were snap‐frozen in liquid nitrogen and dried. Samples were resuspended in 50 mM ammonium bicarbonate, reduced with 2.5 mM DTT for 1 h at room temperature and alkylated with 5 mM iodoacetamide in the dark for 45 min prior to trypsinization. Further processing of samples and identification of proteins was performed as outlined in Marcon et al. (2014). Determination of significant interactors was performed with SAINTexpress using spectral counts and the default settings (Teo et al., 2014).

### 5.7 Ubiquitination assays

In vitro auto‐ubiquitination activity was assayed as outlined in W. Zhang et al., 2016. Assays were performed in 25 μL 50 mM Tris pH 8.0, containing 50 nM E1/UBE1 (Boston Biochem E304), 1 μM E2/UBE2L3, 20 μM ubiquitin (Boston Biochem U100H), 1 μM Smurf E3, and 10 μM UbV. Reactions were incubated at room temperature for 60 min and stopped by the addition of 10 mM EDTA and SDS‐PAGE sample buffer and resolved using 4%–20% gradient gel (Bio‐Rad). Mono‐ and poly‐ubiquitinated Smurf E3s were evaluated by western blotting using the indicated antibody.

Cellular auto‐ubiquitination assays were performed in HEK293 cells transfected with pcDNA3.1 vectors expressing FLAG‐tagged Smurf1/2 and increasing amounts of V5‐tagged UbV, corresponding to a 0.5:1, 1:1 and 2:1 ratio of UbV to transfected Smurf1 or Smurf2. The following day, cells were treated with 40 μM MG‐132 for 2 h and lysed in NP40 lysis buffer (Thermo Fisher) supplemented with HALT™ protease inhibitor cocktail (Thermo Fisher) (Tian et al., 2019). Immunoprecipitation of Smurf was carried out by incubating cell lysates with magnetic FLAG beads (MilliporeSigma; M8823) at 4°C for 2 h. Samples were separated using a NuPage Bis‐Tris 4%–12% gradient gel (Life Technologies) and transferred to a PVDF membrane for western blotting.

For Smad1 ubiquitination assays, HEK293 cells were transfected with Smad1‐HA, FLAG‐Smurf2, and V5‐UbV. The following day, cells were treated with 10 μM MG‐132 for 4 h and subject to immunoprecipitation using anti‐HA antibody. Samples were separated using a NuPage Bis‐Tris 4%–12% gradient gel (Life Technologies) and transferred to a PVDF membrane for western blotting.

### 5.8 Quantitative western blotting

Cellular auto‐ubiquitination assays were performed in triplicate, as previously described in the Ubiquitination Assays section. UbV S2.4 was transfected into HEK293 cells at a 2:1 ratio of UbV S2.4 to Smurf1 or Smurf2. Western blot images were captured to ensure there was no signal saturation prior to quantification in ImageJ (Fiji distribution). All protein bands corresponding to mono‐ and poly‐ubiquitinated species were used for quantification of ubiquitinated Smurf1 and Smurf2, and intensity values were normalized to FLAG‐Smurf1 or Smurf2, V5‐UbV S2.4 and actin input levels. Student's *t* test was used to determine statistical significance between vector and UbV S2.4.

## AUTHOR CONTRIBUTIONS

**Tanner M. Tessier:** Writing – original draft; writing – review and editing; investigation. **Arvid Chowdhury:** Writing – original draft; investigation. **Zane Stekel:** Investigation. **Julia Fux:** Investigation. **Maria Augusta Sartori:** Investigation. **Joan Teyra:** Investigation. **Nick Jarvik:** Investigation. **Jacky Chung:** Investigation. **Igor Kurinov:** Investigation. **Frank Sicheri:** Supervision. **Sachdev S. Sidhu:** Writing – review and editing; supervision. **Alex U. Singer:** Writing – original draft; writing – review and editing; investigation; formal analysis; supervision. **Wei Zhang:** Writing – review and editing; conceptualization; supervision; funding acquisition.

## Supporting information


**Figure S1.** (A) Effect of UbV S2.4 on Smurf1 auto‐ubiquitination. HEK293 cells were transfected with FLAG‐tagged Smurf1 and HA‐tagged wild‐type ubiquitin along with increasing amounts of V5‐UbV S2.4. Cells were treated with 40 μM MG‐132 prior to anti‐FLAG immunoprecipitation to enrich Smurf1. Immunoblotting with anti‐HA antibody was used to determine the level of auto‐ubiquitinated Smurf1 protein. (B) Western blots used for quantification of Smurf1 and Smurf2 auto‐ubiquitination. (C) Smurf2 degrades Smurf1 and is inhibited by UbV S2.4. HEK293 cells were transfected with MYC‐Smurf1, FLAG‐Smurf2 and V5‐UbV S2.4, and whole cell lysates were used to evaluate Smurf1 protein levels.

**Figure S2.** (A) Superposition of the Smurf2 HECT domain comparing structures of the HECT domain alone (PDB 1ZVD) and the HECT domain/UbVS2.4 complex (PDB 7M3Q). Individual subdomains/lobes of the HECT domain are labeled, as well as the position of the UbV. (B) Crystals of the Smurf2 HECT/UbVS2.4 complex following the addition of limiting amounts of trypsin, showing an individual cluster of plates. Individual plates were broken off from this cluster for data collection.
