# Supplementary figures and images for: Structural and functional validation of a highly specific Smurf2 inhibitor
Source: Protein Sci. 2024 Feb 1;33(2):e4885. doi: 10.1002/pro.4885 (PMC10823456; doi:10.1002/pro.4885)

A

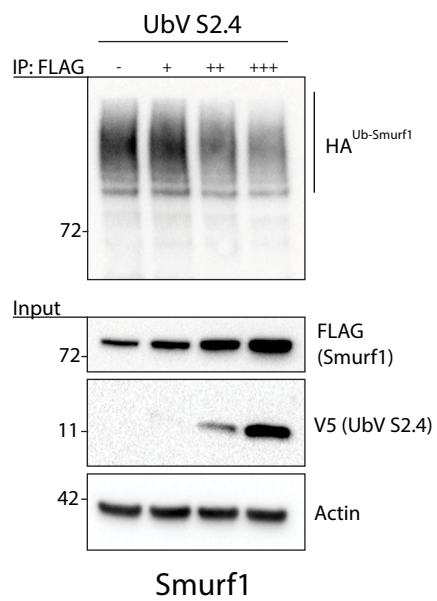

C

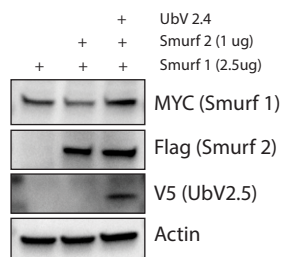

B

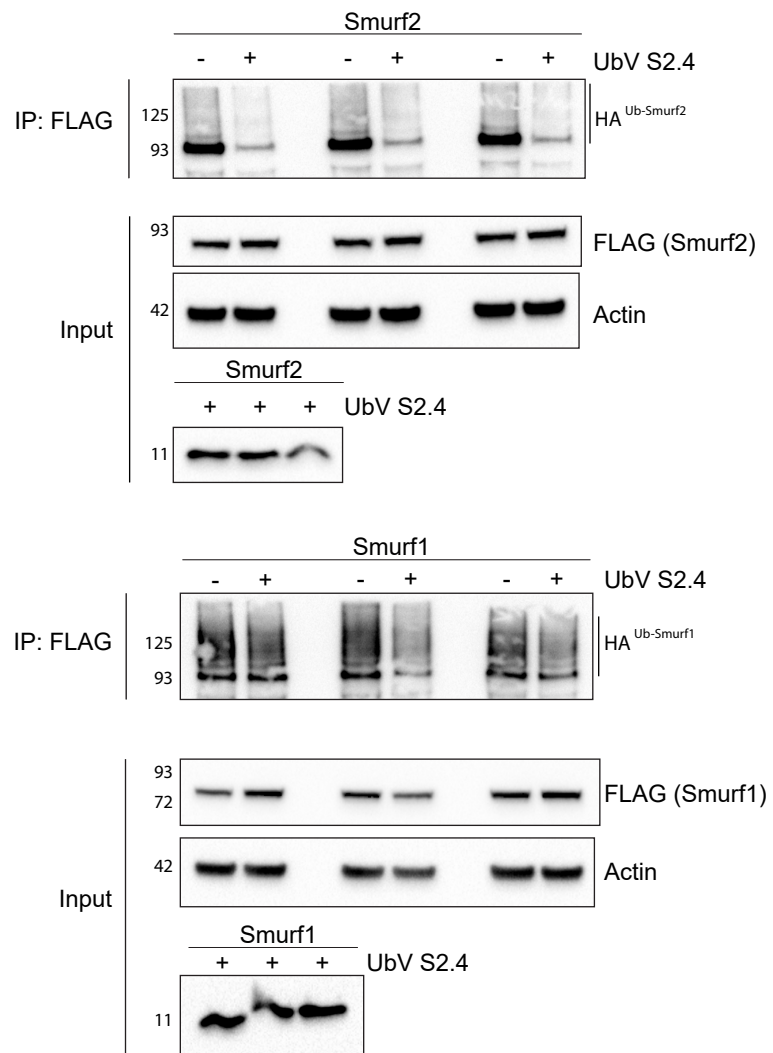

Figure S1

Supplement: Supplementary file 1 — Figure S1. (A) Effect of UbV S2.4 on Smurf1 auto‐ubiquitination. HEK293 cells were transfected with FLAG‐tagged Smurf1 and HA‐tagged wild‐type ubiquitin along with increasing amounts of V5‐UbV S2.4. Cells were treated with 40 μM MG‐132 prior to anti‐FLAG immunoprecipitation to enrich Smurf1. Immunoblotting with anti‐HA antibody was used to determine the level of auto‐ubiquitinated Smurf1 protein. (B) Western blots used for quantification of Smurf1 and Smurf2 auto‐ubiquitination. (C) Smurf2 degrades Smurf1 and is inhibited by UbV S2.4. HEK293 cells were transfected with MYC‐Smurf1, FLAG‐Smurf2 and V5‐UbV S2.4, and whole cell lysates were used to evaluate Smurf1 protein levels. [file PRO-33-e4885-s002.pdf]

A

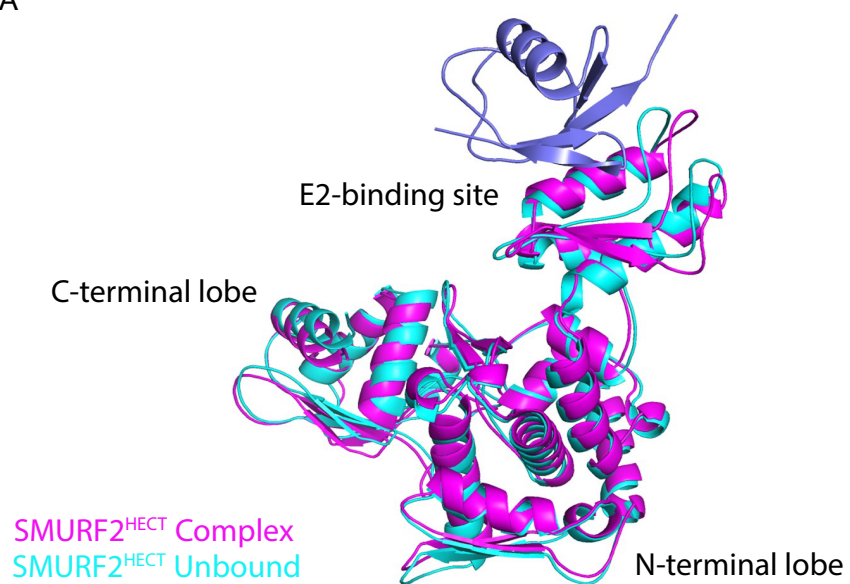

B

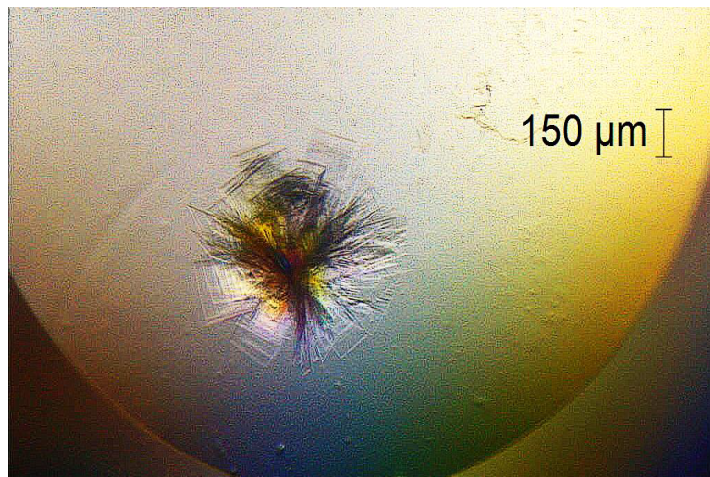

Figure S2

Supplement: Supplementary file 2 — Figure S2. (A) Superposition of the Smurf2 HECT domain comparing structures of the HECT domain alone (PDB 1ZVD) and the HECT domain/UbVS2.4 complex (PDB 7M3Q). Individual subdomains/lobes of the HECT domain are labeled, as well as the position of the UbV. (B) Crystals of the Smurf2 HECT/UbVS2.4 complex following the addition of limiting amounts of trypsin, showing an individual cluster of plates. Individual plates were broken off from this cluster for data collection. [file PRO-33-e4885-s001.pdf]
